# A comparison of retrokeratoprosthetic membrane and conjunctival inflammatory responses to silicone oil

**DOI:** 10.1186/s12348-014-0015-y

**Published:** 2014-06-26

**Authors:** Aubrey L Gilbert, Frederick A Jakobiec, James Chodosh, Dean Eliott

**Affiliations:** 1 David G. Cogan Laboratory of Ophthalmic Pathology, Department of Ophthalmology, Massachusetts Eye and Ear Infirmary, Suit 328, 243 Charles Street, Boston 02114, MA, USA; 2 Cornea and Refractive Surgery Service, Department of Ophthalmology, Massachusetts Eye and Ear Infirmary, 243 Charles Street, Boston 02114, MA, USA; 3 Retina Service, Department of Ophthalmology, Massachusetts Eye and Ear Infirmary, 243 Charles Street, Boston 02114, MA, USA; 4 Department of Ophthalmology, Massachusetts Eye and Ear Infirmary, Harvard Medical School, 243 Charles Street, Boston 02114, MA, USA

**Keywords:** Retrokeratoprosthetic membrane, Granulomatous reaction, Silicone oil, Conjunctiva

## Abstract

Silicone oil continues to be an important aid in retinal detachment surgery. We report a case in which disparate responses to silicone oil were noted in the conjunctiva and intraocularly. Intraocularly, the oil permeated a fibrous membrane that formed behind a keratoprosthesis, the first example of this phenomenon. We detail the histological response to the oil at this site as well as a distinctly different reaction present to oil in the conjunctiva of the same eye. The divergence of histological responses provides a demonstration of the eye's apparent retained capacity to protect against intraocular inflammation, despite multiple previous surgeries.

## Findings

### Summary

Silicone oil continues to be an important aid in the performance of retinal detachment surgery. As complications of its use, we report a 52-year-old man with ocular mucous membrane pemphigoid (MMP) who developed disparate responses to silicone oil in the conjunctiva and intraocularly. The oil permeated a fibrous membrane that formed behind a keratoprosthesis, the first example of this phenomenon.

### Case history

**Figure 1:**
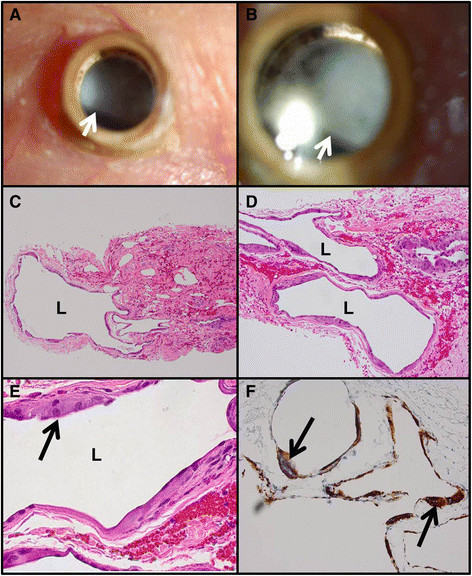
**Clinical photographs and conjunctival histopathology. (A, B)** Clinical photographs of retroprosthetic membrane. The arrows indicate the inferior edge of the membrane. **(C, D)** Multiple locules **(L)** of dissolved-out silicone oil in the conjunctiva display varying sizes. **(E)** Higher power photomicrograph of a locule demonstrating mononucleated, eosinophilic, histiocytic epithelioid cells including a giant cell (arrow). **(F)** CD68 for histiocytes stains the lining cells of the locules. The more intensely stained cells (arrows) are multinucleated giant cells. **(B, D, E,)** Hematoxylin and eosin, ×100, ×200, and ×600. **(F)** Immunoperoxidase reaction, diaminobenzidine chromogen, and hematoxylin counterstain, ×200.

In August 2009, the patient underwent penetrating keratoplasty with implantation of a Boston type II keratoprosthesis (through the eyelid) for extensive corneal and conjunctival cicatrization. Several months later, a fine retroprosthetic membrane had developed and was treated with YAG laser. The patient was then stable until September 2011, when he experienced acutely decreased vision and was found to have vitritis. He underwent a tap and inject procedure with antibiotics and an antifungal agent placed into the eye. The vision improved but a leak around the prosthetic device was identified, prompting a prosthetic replacement. The intraocular pressure was elevated post-operatively, and the patient ultimately underwent an Ahmed valve placement. After the valve was in place, the pressure remained well-controlled, but in January 2012, the patient developed a macula-off retinal detachment for which 1,000 cSt of silicone oil was placed during retinal detachment repair. Subsequently, a new retroprosthetic membrane developed, which became progressively denser over the following months and which was not amenable to treatment with a laser (Figure 1A,B). In August 2012, the patient had surgery for removal of the membrane. At that time, silicone oil from the previous surgery was noted to have migrated into the conjunctiva. Additional samples of conjunctiva containing oil droplets were excised for pathologic examination.

### Results

**Figure 2:**
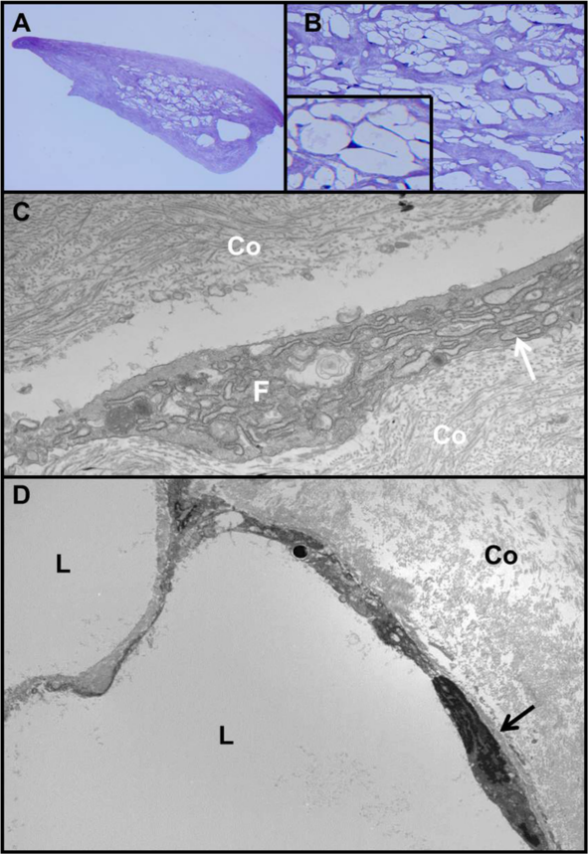
**Retroprosthetic membrane histopathology. (A)** Portion of retroprosthetic fibrous membrane showing myriad vacuoles. Note the absence of a lymphocytic infiltrate. **(B)** Higher power of the spaces in the retroprosthetic membrane. The inset reveals peripherally displaced nuclei and thin strands of compressed cytoplasm. **(C)** Transmission electron micrograph of the fibrous portion of the membrane containing collagen (Co) and fibroblasts **(F)** with profiles of rough-surfaced endoplasmic reticulum (arrow). Note the absence of basement membrane formation around the fibroblast. **(D)** Transmission electron micrograph demonstrating dissolved-out lipid extracellular locules **(L)** and surrounding compressed fibroblasts with stretched-out cytoplasm and flattened nuclei (arrow). Co, membranous collagen. (A) Methylene blue, ×100. **(B)** Methylene blue, ×400; inset, ×600. **(C, D)** Transmission electron micrographs, ×11,000 and ×3,500.

Two distinct histopathologic reactions to silicone oil were observed depending on whether the tissue was intraocular or extraocular. The conjunctival tissue displayed a classic lipogranulomatous response with mononucleated epithelioid cells and multinucleated giant cells surrounding large locules of dissolved-out silicone oil (Figures 1C,D,E). Stains for CD68 (Figure 1F) and CD163 were positive in these histiocytes, while CD1a was negative, establishing that the histiocytes were not Langerhans cells. Review of the retroprosthetic sample revealed smaller extracellular bubbles in the midst of a fibrous membrane (Figure 2A,B). The locules themselves were partially lined by displaced, flattened, or degenerated fibroblastic cells with occasional lipid-like, dense inclusions consistent with silicone oil. No intramembranous histiocytes or other inflammatory cells were discovered as established by electron microscopy (Figures 2C,D).

### Discussion

Histopathologic findings similar to those described for the conjunctival sample above have been noted in prior reports of reactions to silicone oil [[1]]. Although the movement of silicone oil into many other intraocular structures has also been reported, a histiocytic retinal or uveal response has been either absent or severely dampened [[2]], even in eyes enucleated after a decade with oil, except in cases with massive fibrovascular responses [[3]]. There is no earlier published case detailing silicone impregnation of a fibrous retroprosthetic membrane. Such membranes have been shown to arise from corneoscleral stromal downgrowth [[4]], but the potential effect of silicone oil in accelerating or worsening the development of a retroprosthetic membrane must be considered. Some reports have documented the alleged ability of silicone oil to promote the formation of preretinal membranes [[5],[6]]. There is at least one study describing the increased concentrations of fibrogenic growth factors in the setting of intraocular silicone oil [[6]].

One can speculate whether the increased intraocular pressure that the eye experienced may have played a role in forcing silicone oil droplets into the retroprosthetic membrane. The divergence of histological responses to the presence of silicone oil in the conjunctiva versus the retroprosthetic membrane provides a demonstration of the eye's apparent retained capacity to protect against intraocular inflammation, despite multiple previous surgeries.

## Abbreviations

MMP: mucous membrane pemphigoid: 

YAG: yttrium aluminum garnet: 

## Competing interests

The authors declare that they have no competing interests.

## Authors' contributions

AG facilitated the processing and review of all tissue samples, performed the literature review, and drafted the original manuscript. FJ conceived of the paper, reviewed the pathology, and helped to develop the manuscript. JC was one of the surgeons involved in the patient's care, and he provided case history and contributed to the editing of the manuscript. DE was one of the surgeons involved in the patient's care, and he obtained tissue samples, provided case history, and contributed to the editing of the manuscript. All authors read and approved the final manuscript.

## Authors' information

AG is a resident in Ophthalmology. FJ is a former Chief of Ophthalmology and current Director of Ophthalmic Pathology. JC is the Associate Director of the Cornea and Refractive Surgery Service, Director of Boston Keratoprosthesis Clinical Programs, and Director of Education and Fellowship training for the Cornea Service. DE is the Associate Director of the Retina Service.
